# Cancer detection in dogs using rapid Raman molecular urinalysis

**DOI:** 10.3389/fvets.2024.1328058

**Published:** 2024-02-07

**Authors:** John L. Robertson, Nikolas Dervisis, John Rossmeisl, Marlie Nightengale, Daniel Fields, Cameron Dedrick, Lacey Ngo, Amr Sayed Issa, Georgi Guruli, Giuseppe Orlando, Ryan S. Senger

**Affiliations:** ^1^Department of Biomedical Engineering and Mechanics, College of Engineering, Virginia Tech, Blacksburg, VA, United States; ^2^Rametrix Technologies Inc., Blacksburg, VA, United States; ^3^Virginia Maryland College of Veterinary Medicine, Virginia Tech, Blacksburg, VA, United States; ^4^Department of Surgery, VCU Health, Richmond, VA, United States; ^5^Department of General Surgery, Wake Forest University School of Medicine, Winston-Salem, NC, United States; ^6^Department of Biological Systems Engineering, College of Agriculture & Life Sciences and College of Engineering, Virginia Tech, Blacksburg, VA, United States

**Keywords:** Raman spectroscopy, cancer, lymphoma, urine, chemometric, osteosarcoma, mast cell tumor, urothelial carcinoma (UC)

## Abstract

**Introduction:**

The presence of cancer in dogs was detected by Raman spectroscopy of urine samples and chemometric analysis of spectroscopic data. The procedure created a multimolecular spectral fingerprint with hundreds of features related directly to the chemical composition of the urine specimen. These were then used to detect the broad presence of cancer in dog urine as well as the specific presence of lymphoma, urothelial carcinoma, osteosarcoma, and mast cell tumor.

**Methods:**

Urine samples were collected via voiding, cystocentesis, or catheterization from 89 dogs with no history or evidence of neoplastic disease, 100 dogs diagnosed with cancer, and 16 dogs diagnosed with non-neoplastic urinary tract or renal disease. Raman spectra were obtained of the unprocessed bulk liquid urine samples and were analyzed by ISREA, principal component analysis (PCA), and discriminant analysis of principal components (DAPC) were applied using the Rametrix^®^Toolbox software.

**Results and discussion:**

The procedure identified a spectral fingerprint for cancer in canine urine, resulting in a urine screening test with 92.7% overall accuracy for a cancer vs. cancer-free designation. The urine screen performed with 94.0% sensitivity, 90.5% specificity, 94.5% positive predictive value (PPV), 89.6% negative predictive value (NPV), 9.9 positive likelihood ratio (LR+), and 0.067 negative likelihood ratio (LR-). Raman bands responsible for discerning cancer were extracted from the analysis and biomolecular associations were obtained. The urine screen was more effective in distinguishing urothelial carcinoma from the other cancers mentioned above. Detection and classification of cancer in dogs using a simple, non-invasive, rapid urine screen (as compared to liquid biopsies using peripheral blood samples) is a critical advancement in case management and treatment, especially in breeds predisposed to specific types of cancer.

## 1 Introduction

Cancer is common in dogs. According to the American Veterinary Medical Association “Approximately 1 in 4 dogs will, at some stage in their life, develop neoplasia. Almost half of dogs over the age of 10 will develop cancer” (1). Dogs, their owners, and veterinarians, alike, would benefit from tests that differentiate cancer from other disease processes in sick dogs. Likewise, early detection of cancer, when treatment and care might produce better outcomes, would be especially useful in breeds predisposed to a high incidence of specific cancers (e.g., Boxers, Golden Retrievers, Scottish Terriers, German Shepherd Dogs, many others) (2–4).

Recently, several blood sample-based methods for canine cancer detection were described in the literature. One test relies on next-generation genomic sequencing for detection of cancer-associated cell-free DNA fragments in plasma (5, 6). The reported relative observed sensitivity of the blood-based test was 61.5%, with a specificity of 97.5%, and a positive predictive value of 75% for screening patients. A urine-based assay, based on reactive olfactory chemotaxis to volatile organic molecules and other urine molecule by the nematode *Caenorhabditis elegans*, indicated that this nematode could detect cancer in dogs with 90% specificity in a comparison of 48 samples from dogs with cancer and 30 samples from “non-cancer” dogs (7). Other tests and technologies, specifically for detection of canine lymphoma, (plasma microRNA, circulating small extracellular vesicles) have been described in research literature, but are not in general use or commercially-available for cancer detection/management (8, 9).

Urine is a continuously produced “liquid biopsy” of the urinary tract, as well as the entire body. The production and composition of urine is meticulously regulated in health and can be significantly dysregulated in systemic and renal disease (10). Cancer and cancer treatment is known to dysregulate renal structure and function and affect urine composition. This has been recognized in humans for over a century (11) and in dogs since the mid-1960s (12). Cancers localized in the kidney, such as lymphoma and renal cell carcinoma, physically disrupt renal structure and alter renal function as they grow. In humans, malignancies in other organs (lung, colon, prostate)—that may be releasing tumor neoantigens and soluble products—are associated with inflammatory and reactive changes in renal structure and function (13–16); these have been described as malignancy-associated renal paraneoplastic syndromes (17–21). Potentially nephrotoxic chemotherapies clearly can alter renal structure and function during treatment (22–24).

We developed a novel technology—based on precision Raman spectroscopy of urine—that rapidly (within 15 s) distinguishes among molecular vibrations, caused by non-destructive laser irradiation, in hundreds of discrete molecules in urine samples (25–28). The resulting molecular vibration profile is displayed as a spectral image and can be computationally and statistically analyzed, allowing comparison of spectra from healthy individuals and patients with disease, such as cancer.

We have previously reported on this use of a Raman spectroscopy-based technology (Rametrix^®^) (29, 30) for detection of chronic kidney disease (31), diabetic nephropathy (32), renal effects of COVID19 disease (33), microhematuria (34), bladder cancer (35), and chronic Lyme disease (36), validated by comparing Raman spectral patterns in more than 3,000 human patient-derived urine samples and more than 200 healthy human individuals with no clinical or laboratory evidence of renal disease.

We hypothesized in this study that Rametrix® technology would detect metabolic, inflammatory, immune, physiologic (i.e., paraneoplastic) effects of cancer—that alter urine composition. This would create unique “spectral fingerprints” composed of signals from hundreds of molecules present in the urine of dogs with cancer.

Here, we report the results of Raman analysis of urine specimens from 100 dogs with one of four common types of cancer (lymphoma, urothelial carcinoma, osteosarcoma, and mast cell tumor), compared with Raman analysis of 89 clinically healthy dogs and 16 dogs with non-neoplastic urinary tract disease. We demonstrate the detection of cancer-associated multimolecular Raman spectral fingerprints, reflecting local and systemic effects of disease. These results indicate this simple, rapid, noninvasive/minimally invasive method could be used as an aid to the detection and management of cancer in dogs.

## 2 Methods

### 2.1 Study population

Informed written consent for the collection and analysis of urine specimens was obtained from dog owners, following approved Virginia Tech IACUC protocols 15-217, 17-011, and 19-240. A total of 292 dogs were enrolled and urine specimens were collected at the Veterinary Teaching Hospital [VTH] (Blacksburg, VA), Animal Cancer Care and Research Center [ACCRC] (Roanoke, VA), or from study-affiliated referring veterinarians. Referring veterinarians were asked to provide information on whether samples were from generally healthy dogs (based on physical and current laboratory evaluations) or dogs with a confirmed neoplastic disease (cancer) diagnosis. All dogs enrolled from VTH or ACCRC were referred to these hospitals based on prior physical and laboratory evaluations. Of the samples included in this analysis, 100 were from dogs with a cancer diagnosis [Neoplastic (Cancer) group], 89 were from clinically healthy dogs (Healthy group), and 16 from dogs with non-neoplastic urinary tract (UT) disease (UT Disease group) (see Table 1).

**Table 1:** Study population demographics.

Study group	# of patients	Age (years) Range Median	% Male/female	% Male/male neutered	% Female/female neutered
Cancer	100	2–14.67 9.04	51.8/48.2	14.0/86.0	4.0/96.0
Healthy	89	0.2–15 6.67	46.8/50.6	56.4/43.6	50.0/50.0
UT Disease	16	0.3–15 7.34	43.8/56.2	71.4/28.6	44.4/55.6

Histologic confirmation of all tumors was done by board-certified/highly experienced veterinary pathologists associated with veterinary diagnostic laboratories. The expertise in histologic (light microscopic) classification and subtyping of subtle variations in canine lymphoma morphology varied among members of the pathologist cohort. Supplementary immunohistochemical (IHC) staining and flow cytometric (FC) analysis was used to provide additional diagnostic information for some dogs with lymphoma (based on owner participation).

As shown in Table 2, of the 100 dogs with neoplastic cancer included in this study, 53 were diagnosed with canine lymphoma (cL). Histologic/IHC/FC classification of cL subtypes was available for 43/53 patients; 33/43 dogs were classified as having B cell lymphoma, 7 dogs as having T cell lymphoma, and 3 dogs were classified as “mixed histotype”. Thirty-five (35) dogs with cL were enrolled in multiagent chemotherapy protocols; although, none underwent treatment immediately prior to sample collection. Eighteen (18) other dogs with a diagnosis of cL were not undergoing chemotherapy at the time of sample collection. We did not assess whether or how individual drugs or combinations of them may have affected the results of the test. Our study was designed as a first-in-dog pilot of this technology as a screening tool, and assessing the effect of different chemotherapeutics on the performance of the test was not in the scope of this pilot study.

**Table 2:** Urine sample dataset diagnostic classifications.

Urine specimen group (with abbreviation)	Group abbreviation	Number of cases
**Neoplastic diseases (cancer)**	**Cancer**	100
Canine lymphoma	cL	53
*[Lymphoma treated with multiagent chemotherapy]*	cL Chemo	[35]
*[Lymphoma treated with other/no therapies]*	cL No Chemo	[18]
Urothelial carcinoma	UC	18
Mast cell tumor	MCT	17
Osteosarcoma	OS	12
**Non-neoplastic urinary tract diseases**	**UT Disease**	**16**
**Clinically-healthy dogs**	**Healthy**	**89**

Eighteen (18) patients were diagnosed with urothelial carcinoma (UC), 17 were diagnosed with mast cell tumor (MCT), and 12 were diagnosed with osteosarcoma (OS). Of those with MCT, these included cutaneous/visceral tumors that were not distinguished in this study. Likewise, the OS group included appendicular/axial/extraskeletal tumors. Sixteen (16) patients with non-neoplastic urinary tract disease were also included in the study. These included 8 patients with non-tumor bladder disease (cystitis), 5 with kidney stones, and 3 with developmental anomalies, urethral or prostate-associated stricture/retention. Multiple urine samples were obtained from some patients during therapy, but the number of dogs sampled in this manner was insufficient to correlate with efficacy of treatment (i.e., remission or stable disease). This is now being studied with additional cases.

### 2.2 Sample collection and preparation

Urine specimens were collected as midstream “free-catch” voided samples in sterile urine specimen cups or with cystocentesis, as needed. The urine collection methods used represent the standard of care in a veterinary medicine. The goal of our study was to test a novel screening tool for use in real world scenario. We believe that using both urine collection methodologies based purely on clinical need (dogs that would not provide urine via free catch would provide it via cystocentesis) reflects common practice and clinical reality. Urine specimens were stored frozen (−30°C) in sterile vials for no longer than 4 weeks.

To prepare for Raman scanning, samples were thawed in an incubator to 27°C and then approximately 1 mL was pipetted into 1.5 mL screw-top silica glass vials (Thermo Fisher Scientific, Waltham, MA) and sealed. Samples were mixed to suspend any dissolved solids and cellular debris and then Raman spectroscopy was performed on the bulk liquid. The Raman signal was acquired through the side of the glass vial.

### 2.3 Raman spectroscopy

Urine specimens were analyzed by Raman spectroscopy in bulk liquid phase using a PeakSeeker PRO-785 (Agiltron, Woburn, MA) spectrometer equipped with liquid vial holder and fiber optic cable. A 785 nm laser was used with 30s excitation and 30 mW power. The laser spot size was 0.2 mm, and the spectral resolution was 8 cm^−1^. Spectra were collected over the 200–2,000 cm^−1^ wavenumber range, and 10 replicate spectral scans were obtained per sample. RSIQ software (Agiltron) was used to collect spectra and perform initial processing. Surine^™^ Urine Negative Control (Dyna-Tek Industries, Lenexa, KS) was used to align Raman spectra and ensure calibration of the Raman spectrometer.

### 2.4 Chemometric and statistical analysis

Raman spectra were analyzed using the Rametrix^®^ Toolbox v2.0 with MATLAB (R2018a) and the Statistics and Machine Learning Toolbox. The Rametrix^®^ Toolbox v2.0 is available through GitHub and combines elements of the previously published Rametrix^®^ LITE (29) and PRO (30) Toolboxes. The Rametrix^®^ Toolbox was used to read spectral files, average replicates, and truncate spectra to 400–1,800 cm^−1^. It was also used to apply baselining, wavenumber calibration using Surine™, vector normalization, principal component analysis (PCA), discriminant analysis of principal components (DAPC), multivariate analysis of variance (MANOVA), and cross-validation with leave-one-out analysis. Two different baselining methods were applied with the Rametrix^®^ Toolbox. The first was Savitzky–Golay (hereafter abbreviated SG) using a 3^rd^ order polynomial and frame length of 301. The second was ISREA (37), which inserts a cubic spline along defined nodes (knots) of a spectrum. The locations of the nodes were adjusted to improve baseline fit and predictive capabilities. We have applied similar methods in several of our previous studies with human urine (32–34, 36).

### 2.5 Molecular contributions

PCA and MANOVA loading values were used to identify Raman shifts responsible for the clustering separations observed between groups. These loading values were obtained from the PCA and MANOVA procedures performed in MATLAB (using the *pca* and *manova1* functions, respectively). To identify a significant Raman band, a cutoff of 0.3% was used in PCA loadings and 0.2% in MANOVA loadings. These bands were considered significantly different between the groups analyzed, and available Raman libraries (38, 39) were used to determine the biological molecules associated with these bands. We have also applied this approach in other human urine studies and found correlations with available mass spectrometry-based metabolomics data (35, 36).

### 2.6 Cross-validation and screening method development

To validate the modeling described above, a leave-one-out cross-validation method was used. Here, one sample was left-out from the model-building process with PCA, MANOVA, and DAPC. The completed model was then used to predict the group (e.g., cL, Healthy, UC, etc.) of the left-out sample. The prediction was recorded, and the procedure was repeated until every sample in the dataset had been left-out of model-building once. Given the actual and predicted groups of each sample, the overall performance metrics of: prediction accuracy, sensitivity, specificity, positive-predictive value (PPV), and negative-predictive value (NPV) were calculated according to the definitions and formulas published in Trevethan (40). In addition, the resulting urine screen positive and negative likelihood ratios (LR+ and LR−, respectively) were calculated (41) and included as performance metrics.

### 2.7 Study targets for hypothesis testing

This study sought to answer the following questions:

- Can Raman spectroscopy detect differences in the urine metabolomes of dogs with cancer from healthy dogs or those with other non-neoplastic urinary tract disease?
- Can Raman spectroscopy of canine urine be used to distinguish between canine lymphoma (cL), urothelial carcinoma (UC), osteosarcoma (OS), and mast cell tumors (MCT)?
- Does the presence of chemotherapeutic agents impair or influence the urine screen?
- Can Raman spectroscopy of canine urine be used to identify the presence of non-neoplastic urinary tract disease?

**Figure 1:**
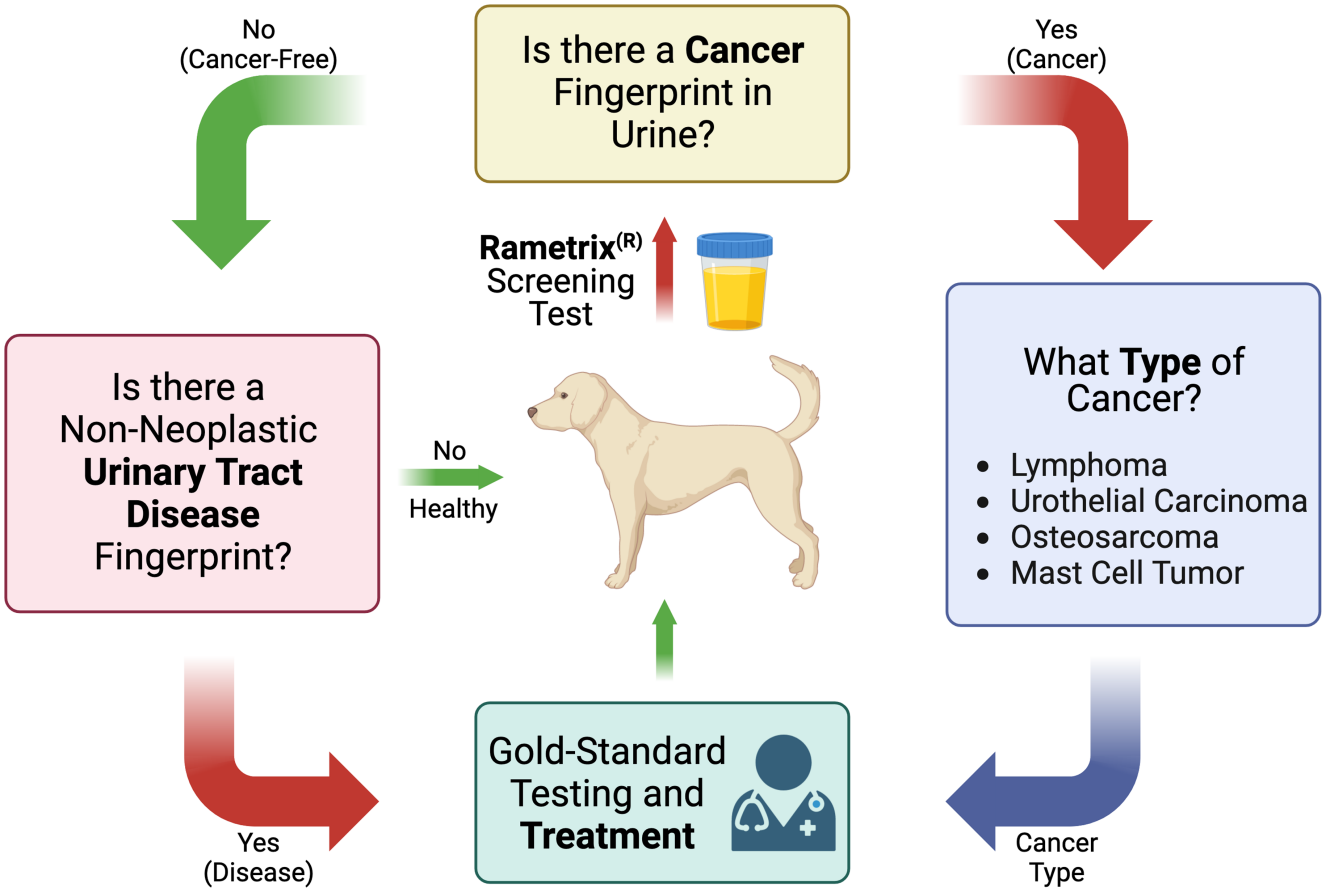
Flow diagram of the Raman spectroscopy-based canine urine screen for cancer, cancer type, and other non-neoplastic urinary tract diseases.

These questions were used to compile the overall screening test flow diagram for canine cancer and non-neoplastic urinary tract diseases shown in Figure 1. It is important to note that the urine screening test described here is intended to supplement established gold-standard testing methods, physical exams, and treatment methods.

## 3 Results

### 3.1 Procedural and computational approach

**Figure 2:**
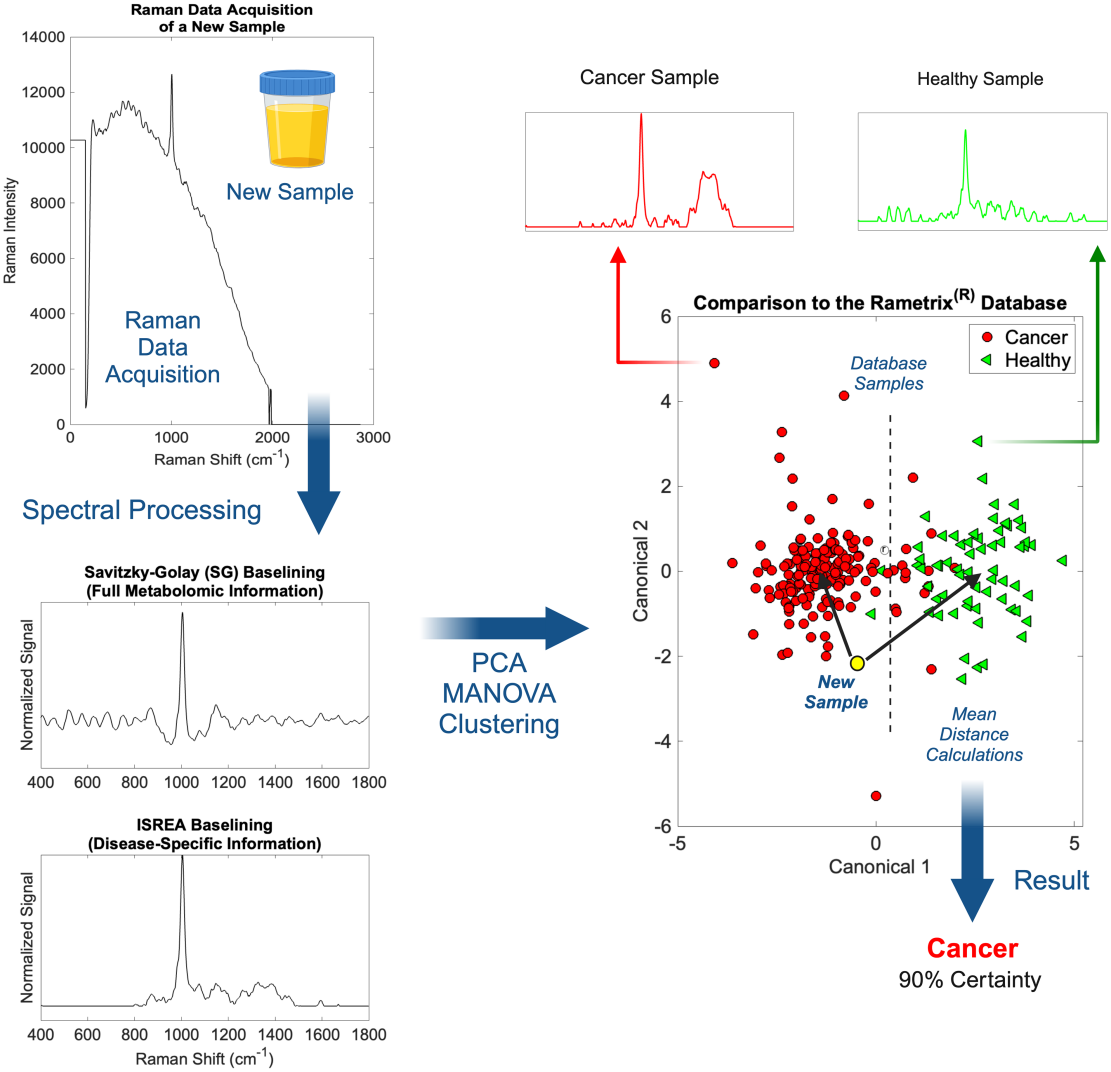
An overview of Raman spectral processing and Rametrix^®^ computations with ISREA and SG baselining.

Scanning of individual urine specimens was accomplished in less than five (5) minutes per sample (multiple laser scans), with no sample preparation required. Urine specimens were aliquoted into 1.5 mL borosilicate glass vials and then scanned. The overall procedure from Raman scanning to obtaining medical information about a urine sample is shown in Figure 2. Once implemented in practice, the spectral processing, baselining, and chemometric calculations associated with Rametrix^®^ were near-instantaneous.

Following the acquisition of a urine Raman spectrum, it was transformed by baselining (Figure 2). This allowed removal of background fluorescence and normalization of the Raman signal. In this study, we used two methods for this: (i) Savitzky–Golay (SG) and (ii) ISREA. SG is a widely known algorithm throughout analytical chemistry, and ISREA is a relatively new development (37). Where SG preserves the entire Raman spectrum, ISREA can amplify regions of spectra containing disease-related information, while minimizing conserved regions. This has served to improve prediction accuracy for spectra of unknown samples in previous studies (32, 33, 36). Results obtained when using ISREA are presented here, and results obtained when using SG are given in the Supplementary material.

Following baselining, chemometric models were produced using PCA, MANOVA, and DAPC. The goal of chemometric modeling was to associate entire spectra (composed of signals from hundreds/thousands of molecules) with a disease condition (e.g., cancer, cL, UC, etc.). In doing this, a spectral “fingerprint” was identified for a specific disease. This has been shown to identify a disease in urine without quantifying all molecules of the underlying metabolome (25, 36, 42–45).

In PCA and DAPC models (Figure 2), each baselined spectrum was reduced to a single data point. Clustering revealed the similarities or differences among the spectra of groups of samples, as with Raman spectroscopy, the spectrum of a sample is representative of its metabolome. Following the building of PCA and DAPC models, the models were cross-validated with leave-one-out analysis, as described previously. Its identity (e.g., a member of the cL group, Healthy group, etc.) was determined and compared to its actual classification. From these calculations, the performance metrics (accuracy, sensitivity, specificity, PPV, NPV, LR+, and LR−) (40, 41) of the Rametrix^®^ urine screening procedure were derived and reported.

### 3.2 Raman spectra

**Figure 3:**
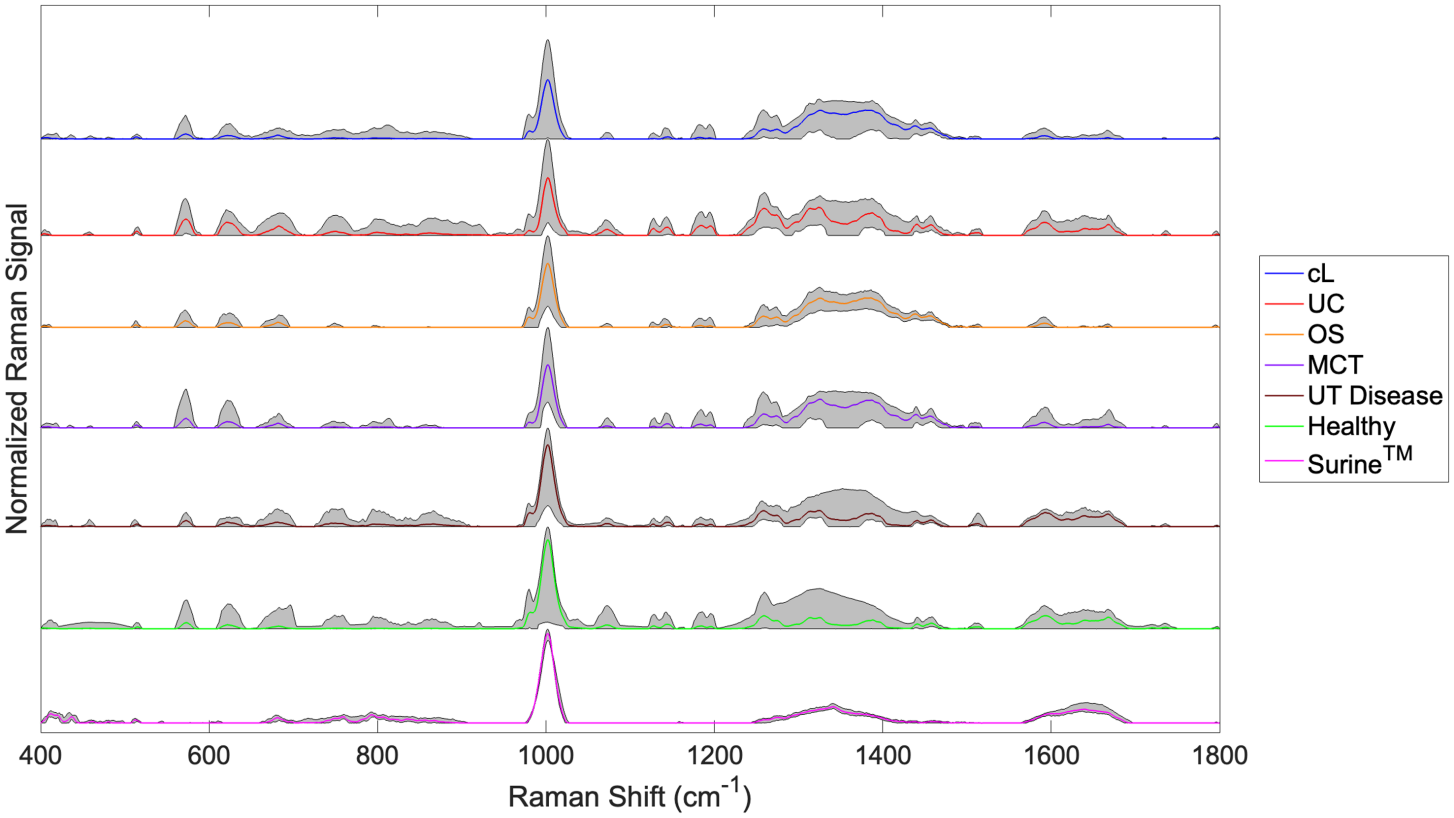
Urine Raman spectra for the canine lymphoma (cL), urothelial carcinoma (UC), osteosarcoma (OS), mast cell tumor (MCT), non-neoplastic urinary tract disease (UT Disease), healthy dogs, and Surine™ groups defined in Table 2. Color lines represent the average group spectrum, and shaded regions show the range of the Raman signal for the group. All spectra were truncated to 400–1,800 cm^−1^, baselined with SG and vector normalized.

Raman spectra for the cL, UC, OS, MCT, UT Disease, and Healthy groups are shown in Figure 3. The spectra for all Surine™ samples analyzed throughout the study are also shown here. In all spectra, color lines represent the average spectrum of the group. The shaded grey region represents the total deviation observed at each Raman shift (or wavenumber). This helps infer a total amount of variability observed among samples. For example, relatively little variability was observed among the 18 samples and scans of Surine™ (Figure 3) acquired. These results affirmed the good working order and calibration of the Raman spectrometer throughout the study.

For samples of the neoplastic (Cancer) disease group (cL, UC, OS, and MCT), a region of relatively high variability was observed in the Raman shift region 1,200–1,400 cm^−1^. This region is routinely dominated by amide Raman bands associated with β -sheet structures and collagen (among others) (38). Upon visual inspection of spectra, many similarities and differences were noted. The chemometric analysis with the Rametrix^®^ Toolbox was used to determine which of these gave rise to unique spectral fingerprints that could be used to determine the identity of an unknown urine specimen. This was done by addressing the four study questions identified earlier.

### 3.3 Can Raman spectroscopy detect differences in the urine metabolomes of dogs with cancer from healthy dogs or those with other non-neoplastic urinary tract disease?

**Figure 4:**
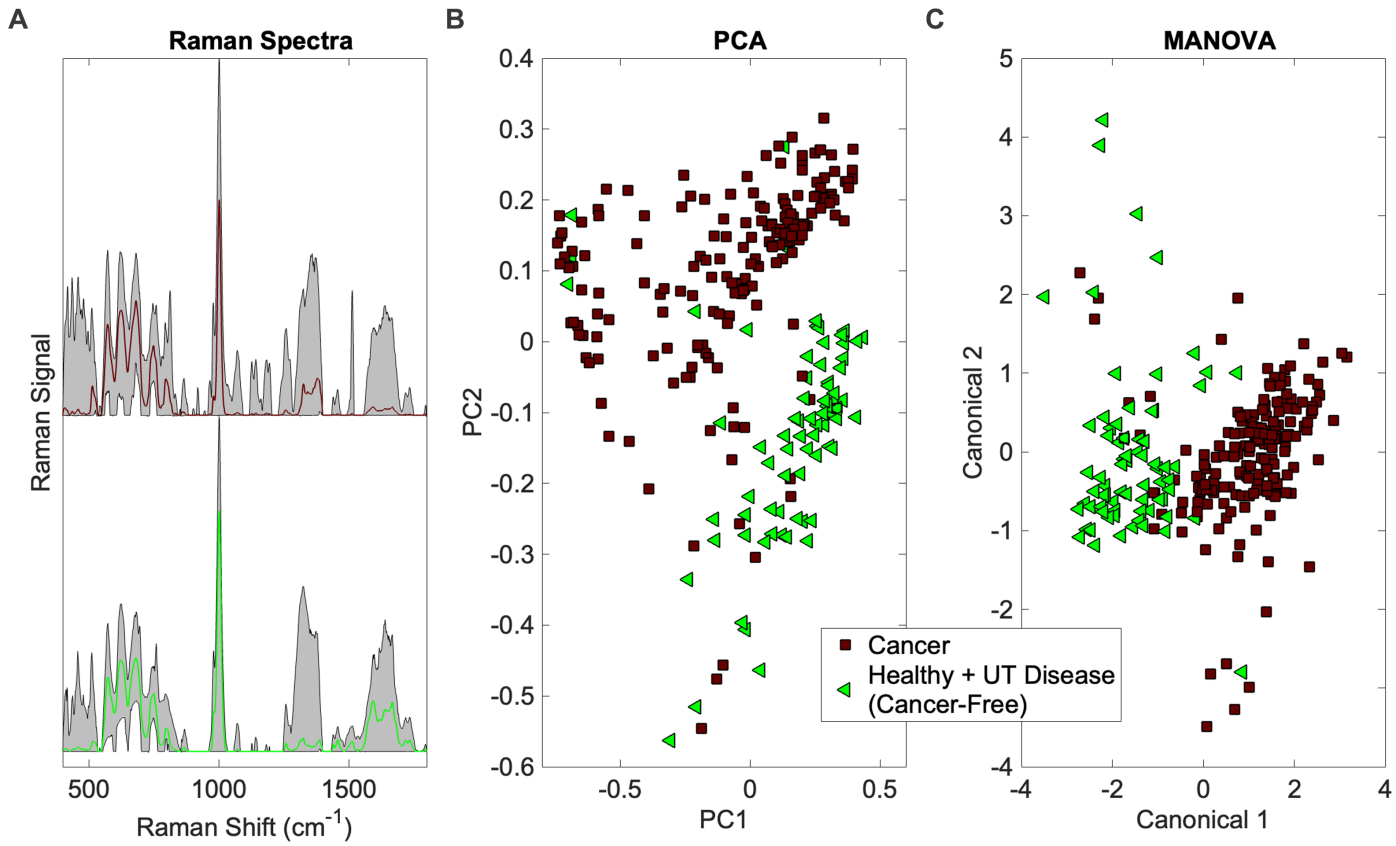
Identification of the cancer spectral signature in canine urine. **(A)** Raman spectra (averaged spectrum in color with total range shaded), **(B)** PCA, and **(C)** MANOVA clustering.

This addresses the first question in Figure 1 (Is there a cancer fingerprint in [canine] urine?). Urine Raman spectra of the Cancer group were compared against the Healthy and UT Disease groups (both cancer-free) using the Rametrix^®^ Toolbox. Chemometric models were built, as shown in Figure 2, to extract a unique spectral fingerprint (s) associated with cancer in dogs. The model was cross-validated with leave-one-out analysis, as described previously, to produce a urine screening model for cancer. The performance metrics (accuracy, sensitivity, specificity, PPV, NPV, LR+, and LR−) are given in Table 3. All percentage-based metrics exceeded 90% (except NPV at 89.6%). The LR+ was 9.9, and the LR− was 0.067. It is noted that an LR+ > 10 and LR− < 0.1 have been identified as “very useful in establishing or excluding a diagnosis” (41). The ISREA nodes used are given in Supplementary Table S1, and performance metrics when using SG are given in Supplementary Table S2. ISREA baselining provided an advantage over SG in extracting a unique spectral fingerprint for cancer from canine urine. For example, the overall accuracy with ISREA was 92.7% (Table 3) and 85.1% with SG (Supplementary Table S2). The plots for ISREA baselined spectra, PCA, and MANOVA testing are shown below in Figure 4. In Figure 4C, the results shown are the predictions when each sample was treated as an unknown, and the remainder of the samples were used to build the predictive model.

**Table 3:** Prediction metrics for urine screens distinguishing Group 1 from Group 2.

Group 1	Group 2	Accuracy (%)	Sensitivity (%)	Specificity (%)	PPV (%)	NPV (%)	LR+ (Group 1)	LR− (Group 2)
Cancer	UT Disease + Healthy	92.7	94.0	90.5	94.5	89.6	9.9	0.067
cL	UC + OS + MCT	71.0	69.8	72.3	74.0	68.0	2.5	0.42
UC	cL + OS + MCT	94.0	83.3	96.3	83.3	96.3	22.5	0.17
OS	cL + UC + MCT	92.0	58.3	96.6	70.0	94.4	17.1	0.43
MCT	cL + UC + OS	81.0	52.9	86.8	45.0	90.0	4.0	0.54
cL	Healthy	100	100	100	100	100	Inf*	0
UC	Healthy	95.3	88.9	96.6	84.2	97.7	26.1	0.11
OS	Healthy	100	100	100	100	100	Inf	0
MCT	Healthy	98.1	88.2	100	100	97.8	Inf	0.12
cL (No Chemo)	cL (Chemo)	73.6	82.8	55.6	78.4	62.5	1.86	0.31
UT Disease	Healthy	93.3	68.8	97.8	84.6	94.6	31.2	0.32

*Inf, infinite due to division by zero.

The PCA and MANOVA loadings were also investigated to determine which Raman bands were responsible for comprising the cancer fingerprint in canine urine. Here, the top 27 loadings were investigated. Of these, 11 were associated with Raman bands present in both PCA and MANOVA loadings. One was assigned, according to Talari et al. (38) and Movasaghi et al. (39), to phosphatidylinositol (576 cm^−1^) and six were associated with protein (notably collagen) (621, 1,011, 1,160, 1,260, 1,313, 1,344 cm^−1^). In addition, two bands were associated with fatty acids and lipids (1,300 and 1,313 cm^−1^), one with glucose (1,344 cm^−1^), and three bands were undefined (685, 983, 1,644 cm^−1^). Notable among the MANOVA loadings included assignments for red blood cells (991 cm^−1^) (46) and malignant tissues (1,450 cm^−1^) (47, 48).

### 3.4 Can Raman spectroscopy of canine urine be used to distinguish between canine lymphoma (cL), urothelial carcinoma (UC), osteosarcoma (OS), and mast cell tumors (MCT)?

Next, we sought to determine if Rametrix^®^ could distinguish between the different cancer types. This is shown in Figure 1, where cancer type would be determined should a sample screen positive for cancer (see the previous section). To do this, a particular cancer group (e.g., cL) was analyzed against the combination of the other cancer groups (e.g., UC + OS + MCT) do determine if a unique spectral fingerprint existed for that particular cancer. Results are shown in Table 3 (and in Supplementary Table S2 for SG baselining). UC was the most recognizable cancer, with all prediction metrics exceeding 83%. As outlined in Trevethan (40) and Ranganathan and Aggarwal (41), the suitability of a screening method is determined by all of its percentage-based performance metrics (accuracy, sensitivity, specificity, PPV, and NPV) as well as LR+ and LR−. Thus, when considering a Rametrix^®^ urine screen for UC, we do so by the minimum percentage-based metric (sensitivity and PPV of 83.3%) even though other metrics (specificity and NPV) achieved 96.3%. This helps evaluate cases, such as OS, which had a similar overall accuracy to UC (92.0% vs. 94.0%) but had a minimal metric of 58.3% (sensitivity). This is also apparent in LR+ and LR− values, but these calculated values incorporate sensitivity and specificity only. Thus, the screening test for UC was considered superior to that of OS. This is likely a reflection of the direct interaction of urothelial carcinoma and cancer associated pathology (local inflammatory reaction and bleeding) with urine sampled. The screen for cL had a minimal metric of 68.0%, and that for MCT had a minimal metric of 52.9%.

The individual cancers were also tested against the Healthy group. These results are also given in Table 3 and Supplementary Table S2. Here, the minimal metrics for cL, UC, OS, and MCT were 100%, 84.2%, 100%, and 88.2%, respectively. This suggests that when screening for one particular type of cancer, in the absence of any non-neoplastic UT diseases, more effective urine screens exist.

### 3.5 Does the presence of chemotherapeutic agents impair or influence the urine screen?

It was necessary to show that chemotherapeutic agents in the urine of cancer patients were not responsible for the cancer spectral fingerprint detected earlier. To verify our result, we compared the cL Chemo group (cL patients receiving chemotherapeutics) to the cL No Chemo group (cL patients receiving treatment not involving chemotherapeutics). Again, the prediction metrics of Rametrix^®^ urine screen are given in Table 3 and Supplementary Table S2. As shown previously, the minimal metric for cancer detection was 89.6% (NPV). In distinguishing the groups cL Chemo from cL No Chemo, the minimal metric was 55.6% (specificity). In addition, the overall accuracies of the screens were 92.7% and 73.6% for cancer and chemotherapeutic detection, respectively. Furthermore, spectral plots (see Supplementary Figure S1) appear nearly identical for the cL Chemo and cL No Chemo groups. Because of this, we conclude cL (and likely other cancers) has/have a spectral fingerprint that is independent of chemotherapeutics (or their break-down products) that might reside in the urine. This clearly will require further study with a larger number of dogs undergoing sequential urine collection during chemotherapy.

The PCA and MANOVA loadings were also investigated to learn which Raman bands are significant in separating the cL No Chemo and Healthy groups. Unique Raman bands were sought that did not appear in the previous cancer fingerprint. There were 10 unique loadings investigated. Of these, four were related to nucleic acids (particularly cytosine) (1,287, 1,293, 1,325, 1,423 cm^−1^). Three were related to protein (700, 1,602, 1,660 cm^−1^), and one was related to porphyrin (1,620 cm^−1^) (49). The malignant tissue band (1,450 cm^−1^) was recognized in this analysis. In addition, numerous bands were recognized in the cL spectral signature that coincided with the general cancer signature discussed earlier.

### 3.6 Can Raman spectroscopy of canine urine be used to identify the presence of non-neoplastic urinary tract disease?

As shown in Figure 1, if a urine specimen screened negative for cancer, it would be screened further for the presence of non-neoplastic urinary tract disease. Thus, the UT Disease group was analyzed against the Healthy group to determine if a UT Disease fingerprint was present. We note that the Cancer group is not included here, as cancer is screened first and given top priority in our screening model. Results are given in Table 3 and Supplementary Table S2. The overall accuracy for detecting UT disease, such as uroliths and inflammatory/infectious cystitis was 93.3%, with a minimal metric of 68.8% (sensitivity), LR+ of 31.2, and an LR− of 0.32. Thus, the screen for a cancer fingerprint produced better overall metrics than that for a non-neoplastic urinary tract disease given the dataset analyzed.

## 4 Discussion

A simple, rapid, and inexpensive urine screening test, comparable in cost ($75–$150) to routine hematology/chemistry/urinalysis testing (individually), to identify the presence of cancer, using dog urine, should be of significant interest to dog owners, breeders, and veterinarians. Individual pet owners clearly want to enjoy the many benefits of their pets and have a sincere, well-founded interest in maintaining their dog’s health. Many/most are aware that neoplastic disease is common in middle-aged and older dogs, and owners of some “high risk breeds” (Golden Retrievers, Boxers, for example) are acutely aware of the ever-present reality of cancer with the advancing age of their pets. Most dog owners and their veterinarians understand that, in many cases, early detection of cancer is potentially correlated with better outcomes in terms of tumor control and quality of life. By the same token, when presented with dogs that have cancer, owners and their veterinarians face difficult choices, including whether to treat and how they can evaluate if treatment is working. Our current tools (clinical observation, imaging, and laboratory studies) are, at best, imprecise (and expensive) for determining treatment efficacy. The method described here is designed to inform veterinarians using a rapid and inexpensive urine screen that could be applied broadly. Veterinarians will likely choose to supplement these results with further testing, including imaging, biopsy, and other laboratory tests. We are not proposing to replace any gold-standard methods or accepted practices.

Here, we have shown that our approach with Raman spectroscopy and Rametrix^®^ chemometric modeling may be able to fill gaps and to provide a readily-accessible, simple, and accurate method for cancer detection. In this “first-in-dog” pilot study, when testing with a Cancer group consisting of 100 samples representing four common cancers in canines, the broad spectral fingerprint of cancer was detected with better than 92% accuracy (with 94% sensitivity, 90% specificity, LR+ of 9.9, and LR− of 0.067).

The resulting canine urine screens could be implemented in practice as follows. Each screen (e.g., Cancer vs. UT Disease + Healthy in Table 3) consists of a set of ISREA nodes, a PCA coefficient matrix, a PCA vector of mean values, a MANOVA eigenvector matrix, and a logic gate to separate clusters by canonical scores (Figure 4C). These numerical values comprise the Rametrix^®^ screening model, and they can be delivered to remote Raman scanners. Thus, large datasets of Raman spectra of canine urine only need to be compiled in one centralized location. Once installed, the resulting Rametrix^®^ screening model will enable screening of a locally obtained urine sample against our large database of samples. As our database continues to grow, the Rametrix^®^ screening model will be updated and distributed to Raman scanners (either at a commercial veterinary pathology laboratory service or at veterinary practices) easily. We also have developed methods to calibrate and synchronize remotely deployed scanners and have even developed custom Raman devices to automate scanning multiple urine samples and provide Rametrix^®^ screening model results. Implementation of this long-term vision is a future endeavor beyond the scope of the study described in this paper though.

From a data modeling perspective, we observed in this study an advantage from ISREA baselining over an industry-standard SG method. We highlighted the comparison of these two methods because ISREA remains relatively new and has appeared in a limited number of our recent publications of results analyzing human patient urine specimens. SG, on the other hand, has been well established in signal processing and analytical chemistry for many years. We would question results if discernible differences and cancer fingerprints were detected with ISREA but absent with SG baselining. Here, we observed the identification of fingerprints with both methods but saw seemingly significant advantages from ISREA, making it a necessary inclusion in the Rametrix^®^ screening model. In addition, this method enabled us to identify numerous Raman bands (and associated biomolecules) that differ in the urine of dogs with cancer and healthy dogs. Thus, the identification of cancer in urine was done here by the recognition of spectral signatures from several molecules, instead of a singular cancer biomarker. In addition, the signature for cL shared many similarities to the general cancer signature; however, unique characteristics were noted for cL.

There are limitations of this study that affect interpretation and broad generalizations of the results:

- The Cancer group of this study consisted of samples from dogs with cL, UC, MCT, and OS. These were included because they were most commonly referred to us for evaluation and treatment; thus, they were most represented in our larger dataset. In future studies, the Cancer group will contain samples from dogs with several other neoplastic diseases.
- Although more than adequate for a proof-of-concept of the Raman spectral fingerprinting methodology, samples were obtained from a relatively small and heterogenous group of cancer cases. For example, differences among appendicular/axial/extraskeletal tumors were not sought among the OS group due to small sample numbers in the dataset. This will be remedied by ongoing case accrual and analysis in coming years. Another example is the UT Disease group in this study. This group was under-represented in patient numbers relative to the Cancer and Healthy groups. We hypothesize that with a larger and more well-defined population, the detection of non-neoplastic urinary tract disease with our screen will improve. We readily acknowledge that we need to do additional research on spectral fingerprints associated with chronic renal disease, a very common morbidity in middle-aged and older dogs (50–57).
- It is possible that some dogs that were clinically healthy may have had undiagnosed neoplastic disease or other evolving co-morbidities. Future studies will include provisions for continuing contact with dog owners and their veterinarians to define the context of “healthy” at the time of sampling. In fact, one realistic and desirable use of this Raman spectroscopy-based technology may be periodic screening of healthy dogs for undiagnosed neoplastic disease (i.e., early detection through screening). Based on the results presented here, however, we cannot determine if our methods are sensitive enough to detect incipient neoplastic disease. We are committed, in planned studies, to see if early detection is indeed possible.
- We acknowledge that approximately 95% of urine samples were collected by voided “free-catch,” while the others were collected from catheters or cystocentesis. Again, the sample numbers were too small in this proof-of-concept study to probe the collection method in detail. No significant differences were noted in spectra by collection method by visual inspection. In addition, no significant differences were observed for voided vs. catheter urine collections from human patients in prior studies (33, 58).
- We did not document, with serial sampling, the effects of antineoplastic treatments for dogs on this study. A few dogs, under treatment, did provide multiple samples both during and at the conclusion of treatment (due to remission, withdrawal from study, or disease progression). However, the number of dogs for which we had serial sampling data was insufficient to draw meaningful conclusions. Serial sampling during treatment will be written into future sampling protocols and studies, especially studies we intend to conduct on lymphoma, where we hypothesize that there would be changes in the Raman urine molecular fingerprint correlated to disease remission, stabilization, or conversely, to progression and treatment failure.
- We readily acknowledge that the results of our Raman spectroscopy-based molecular urinalysis must be kept and used in a clinical context of history, physical examination, concurrent laboratory and imaging studies, and patient observation. It would be inappropriate and ill-advised to have a positive (cancer positive) Raman urine spectral fingerprint trigger expensive and perhaps needless testing—or to be used to be a definitive, single metric for deciding whether or not to euthanize a dog.

We hope the novel technology described here can be made available—for the benefit of dogs, their owners, breeders, and veterinarians, alike through distributed urine Raman scanning devices and Rametrix^®^ screening model. To be of real value in managing cancer in dogs, much future work needs to be done.

## 5 Conclusion

An accurate and rapid urine screening test for detecting cancer in dogs is presented here. The assay uses Raman spectroscopy to discern spectral fingerprints—composed of hundreds of molecules (*not* single biomarkers)—in urine. It could be used as part of regular wellness evaluations—as an aid in early/earlier detection of cancer, especially in breeds highly predisposed to development of certain types of cancer. It may also be useful in assessing responses to therapy and potentially in evaluating recurrence or progression of tumors. Although in this study, we focused on lymphoma, the most common hematologic cancer in dogs, and on other common and serious malignancies (urothelial carcinoma, mast cell tumor, and osteosarcoma), we intend to expand our studies to dogs of all ages and breeds, including those suffering from other malignancies. Major advantages of the approach presented here are that it is inexpensive, rapid, and requires minimal sample preparation. The numerous molecules of the cancer spectral fingerprint can be measured in a single assay.

## Data availability statement

The raw data supporting the conclusions of this article will be made available by the authors, without undue reservation.

## Ethics statement

The animal studies were approved by Virginia Tech IACUC protocols 15-217, 17-011, and 19-240. The studies were conducted in accordance with the local legislation and institutional requirements. Written informed consent was obtained from the owners for the participation of their animals in this study.

## Author contributions

JLR: Conceptualization, Funding acquisition, Investigation, Methodology, Project administration, Resources, Supervision, Validation, Visualization, Writing – original draft, Writing – review & editing. ND: Conceptualization, Investigation, Methodology, Resources, Supervision, Validation, Writing – review & editing. JR: Investigation, Methodology, Resources, Supervision, Validation, Writing – review & editing, Data curation. MN: Investigation, Methodology, Resources, Writing – review & editing. DF: Investigation, Data curation, Writing – review & editing. CD: Data curation, Investigation, Writing – review & editing. LN: Data curation, Investigation, Writing – review & editing. AI: Data curation, Investigation, Formal analysis, Methodology, Software, Supervision, Writing – review & editing. GG: Conceptualization, Validation, Writing – review & editing. GO: Conceptualization, Validation, Writing – review & editing. RS: Conceptualization, Validation, Data curation, Formal analysis, Funding acquisition, Investigation, Methodology, Project administration, Resources, Software, Supervision, Visualization, Writing – original draft, Writing – review & editing.
