# Supplementary material for: Cancer detection in dogs using rapid Raman molecular urinalysis
Source: Front Vet Sci. 2024 Feb 7;11:1328058. doi: 10.3389/fvets.2024.1328058 (PMC10879274; doi:10.3389/fvets.2024.1328058)
Supplement: Supplementary file 1 [file Data_Sheet_1.pdf]

Supplementary Appendix

Cancer detection in dogs using rapid Raman molecular urinalysis

John L. Robertson[1,2], Nikolas Dervisis[3], John Rossmeisl[3], Marlie Nightengale[3], Daniel Fields[3], Cameron Dedrick[3], Lacey Ngo[1], Amr Sayed Issa[2], Georgi Guruli[4], Giuseppe Orlando[5], Ryan S. Senger[2,6]

[1] Department of Biomedical Engineering and Mechanics, College of Engineering, Virginia Tech, Blacksburg VA 24061
[2] Rametrix Technologies Inc., 2000 Kraft Drive Suite 1208, Blacksburg VA 24060
[3] Virginia Maryland College of Veterinary Medicine, Virginia Tech, Blacksburg VA 24061
[4] Department of Surgery, VCU Health, Richmond, VA 23219
[5] Department of General Surgery, Wake Forest University School of Medicine, Winston-Salem, NC 27101
[6] Department of Biological Systems Engineering, College of Engineering, Virginia Tech, Blacksburg VA 24061

Corresponding Authors:
John L. Robertson: drbob@vt.edu
Ryan S. Senger: senger@vt.edu

Table S1. Detection of spectral fingerprint in an unknown dog urine sample to discern Groups 1 and 2 using the Rametrix® DAPC cancer model with Savitzky-Golay spectral baselining.

Group 1	Group 2	Number of PCs Used	% Dataset Variance	Accuracy	Sensitivity	Specificity	PPV	NPV
LSA	Control	7	94.2%	98.3%	96.23%	99.2%	98.1%	98.4%
BCA	Control	20	98.9%	90.8%	55.6%	95.9%	66.7%	93.7%
LSA	BCA	9	96.5%	88.7%	96.2%	66.7%	89.5%	85.7%
BCA	Human BCA	17	99.4%	91.4%	88.9%	94.1%	94.1%	88.9%
LSA Chemo	LSA No Chemo	5	93.2%	66.0%	82.3%	33.3%	70.7%	50.0%
LSA No Chemo	Control	5	91.5%	94.4%	100%	88.9%	90.0%	100%

Table S2. ISREA nodes designed for each case study.

Group 1	Group 2	ISREA Nodes
Cancer	Control	400, 796, 819, 1508, and 1800 cm^{-1}
LSA	Control	400, 679, 972, 1181, 1618, 1800 cm^{-1}
BCA	Control	400, 425, 443, 684, 1593, 1800 cm^{-1}
LSA	BCA	400, 838, 951, 1354, 1654, 1800 cm^{-1}
BCA	Human BCA	400, 437, 601, 649, 1634, 1800 cm^{-1}
LSA Chemo	LSA No Chemo	400, 608, 617, 920, 1147, 1800 cm^{-1}
LSA No Chemo	Control	400, 1300, 1434, 1696, 1800 cm^{-1}

Note: Groups 1 and 2 are defined in Table 1 of the publication.

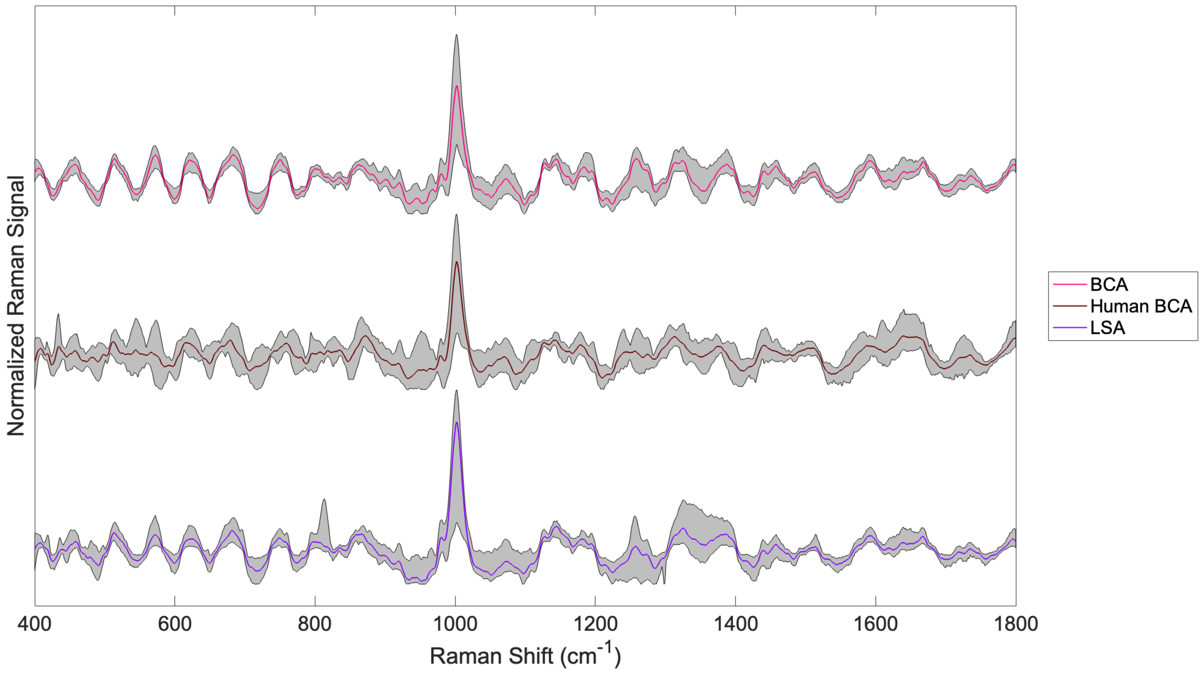

Fig. S1. Urine Raman spectra for the BCA, Human BCA, and LSA groups defined in Table 1. All spectra were truncated to 400-1,800 cm^{-1}, baselined with Savitzky-Golay, and vector normalized.

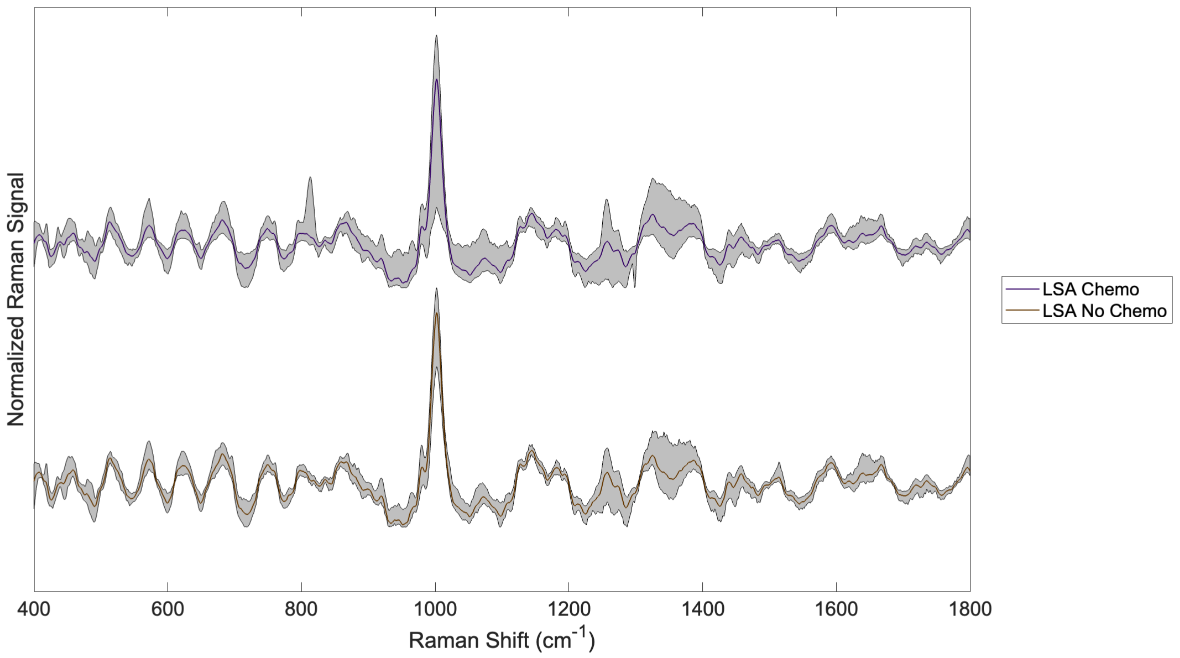

Fig. S2. Urine Raman spectra for the LSA Chemo and LSA No Chemo groups defined in Table 1. All spectra were truncated to 400-1,800 cm^{-1}, baselined with Savitzky-Golay, and vector normalized.
